# The role of neighborhood level socioeconomic characteristics in *Salmonella *infections in Michigan (1997–2007): Assessment using geographic information system

**DOI:** 10.1186/1476-072X-6-56

**Published:** 2007-12-19

**Authors:** Muhammad Younus, Edward Hartwick, Azfar A Siddiqi, Melinda Wilkins, Herbert D Davies, Mohammad Rahbar, Julie Funk, Mahdi Saeed

**Affiliations:** 1 Department of Epidemiology, Michigan State University, East Lansing, Michigan 48824, USA; 2 National Food Safety and Toxicology, Michigan State University, East Lansing, Michigan 48824, USA; 3 Large Animal Clinical Sciences, Michigan State University, East Lansing, Michigan 48824, USA; 4 Communicable Disease Division, Michigan Department of Community Health, 201 Townsend Street, Lansing, Michigan 48913, USA; 5 Departments of Pediatrics and Human Development, Michigan State University, East Lansing, Michigan 48824, USA

## Abstract

**Background:**

The majority of U.S. disease surveillance systems contain incomplete information regarding socioeconomic status (SES) indicators like household or family income and educational attainment in case reports, which reduces the usefulness of surveillance data for these parameters. We investigated the association between select SES attributes at the neighborhood level and *Salmonella *infections in the three most populated counties in Michigan using a geographic information system.

**Methods:**

We obtained data on income, education, and race from the 2000 U.S. Census, and the aggregate number of laboratory-confirmed cases of salmonellosis (1997–2006) at the block group level from the Michigan Department of Community Health. We used ArcGIS to visualize the distribution, and Poisson regression analysis to study associations between potential predictor variables and *Salmonella *infections.

**Results:**

Based on data from 3,419 block groups, our final multivariate model revealed that block groups with lower educational attainment were less commonly represented among cases than their counterparts with higher education levels (< high school degree vs. ≥ college degree: rate ratio (RR) = 0.79, 95% confidence interval (CI):0.63, 0.99; ≥ and high school degree, but no college degree vs. ≥ college degree: RR = 0.84, 95% CI: 0.76, 0.92). Levels of education also showed a dose-response relation with the outcome variable, i.e., decreasing years of education was associated with a decrease in *Salmonella *infections incidence at the block group level.

**Conclusion:**

Education plays a significant role in health-seeking behavior at the population level. It is conceivable that a reporting bias may exist due to a greater detection of *Salmonella *infections among high education block groups compared to low education block groups resulting from differential access to healthcare. In addition, individuals of higher education block groups who also have greater discretionary income may eat outside the home frequently and be more likely to own pets considered reservoirs of *Salmonella*, which increase the likelihood of contracting *Salmonella *infections compared to their counterparts with lower levels of education. Public health authorities should focus on improving the level of disease detection and reporting among communities with lower income and education and further evaluate the role of higher educational attainment in the predisposition for salmonellosis.

## Background

*Salmonella *serotypes are a major cause of global foodborne infections and are among the leading causes of foodborne illness. Additionally, *Salmonella *serotypes are considered as the second most frequent cause of bacterial diseases in the United States (U.S.) [1]. Despite efforts to reduce disease burden associated with *Salmonella *infections including improved sanitation and safety, salmonellosis remains a major public health problem in the U.S. [2,3]. *Salmonella *infections (salmonellosis) accounts for an estimated 1.4 million cases of illness, including over 100,000 physician office visits [4], 16,000 hospitalizations, and 600 deaths each year [5].

Efforts to reduce the burden of the disease rely to a great extent on a clear understanding of its determinants in the population. Findings from observational epidemiologic investigations have resulted in the identification of certain demographic attributes associated with infectious diseases including salmonellosis, such as extremes of age [6,7], gender, and overcrowding [8]. However, individuals' socioeconomic status (SES) (e.g., education attainment, household income, and employment status,) that is recognized as an important determinant of certain chronic diseases and conditions [9-12] (e.g., cardiovascular disease, diabetes, and cancer) has not been extensively evaluated for its role in the incidence of infectious diseases, particularly *Salmonella *infections. Limited evidence exists to explain the influence of SES on the incidence and distribution of infectious agents in susceptible populations. A few studies, conducted mainly outside the U.S., suggest that economic deprivation at the individual, household, and community level increases the likelihood of experiencing certain bacterial [8], parasitic [13], and viral infections [14]. Despite sharing many common features, infectious diseases are not the same, and while useful, the findings of these studies may not apply equally to all infectious diseases, leaving a need for detailed studies on individual diseases. This research is an attempt to study the association between the incidence of *Salmonella *infections and neighborhood level socioeconomic attributes, particularly income and education.

Incomplete information in case reports regarding individual level socioeconomic indicators like household or family income, education attainment, and employment status in the majority of U.S. disease surveillance systems reduces the usefulness of surveillance data for these parameters [15,16]. In addition to socioeconomic data, the non-mandatory disclosure of racial and ethnic information during the disease investigation process, for both notifiable and non-notifiable diseases or conditions, also adds to the problem of incomplete information on these characteristics, restricting their evaluation as potential risk factors. Our recent work [17,18] examining the association between demographic variables and *Salmonella *infections in Michigan revealed that a substantial proportion (43%) of laboratory-confirmed case reports of salmonellosis in the statewide Michigan Disease Surveillance System (MDSS) did not contain race and ethnicity information, thus limiting our ability to study disease variation among racial and ethnic groups. From a public health standpoint, understanding the interplay of race with other factors associated with a given disease or condition is very important because reducing health disparities remains a challenging and current issue for public health officials. In fact, the Healthy People 2010 initiative has declared the reduction of racial disparities a primary public health goal [19].

Investigating the association between socioeconomic indicators and *Salmonella *infections, including effects on the distribution of *Salmonella *cases in the population, may be of limited value from perspective of disease causation and prevention at the individual level, but its true value lies from a disease control and prevention standpoint where it can help guide state and federal public health officials in determining the focal points of their interventions. Additionally, identification of group level risk factors for any given disease or condition using Geographic Information System (GIS) technology is certainly less expensive than traditional data collection techniques. It could prove to be a more cost-effective approach to disease prevention than focusing on individual level factors.

GIS technology, along with area-based socioeconomic measures (ABSMs) gathered by the U.S. Bureau of Census during decennial censuses, could provide a potential solution in the absence of individual level socioeconomic data for studying the relation between these variables and the distribution of disease at the neighborhood or group level. GIS is a geographic mapping and analysis tool, and is capable of integrating large geographic data and linking it to non-geographic data (e.g., socio-demographic characteristics) [20,21]. We carried out this study using GIS technology to identify the associations between neighborhood-based socioeconomic characteristics including education, income and race and *Salmonella *infections in Michigan between 1997 and 2006.

## Methods

### Study design and settings

This study is an ecological analysis of the neighborhood-level socioeconomic and demographic factors and reported cases of salmonellosis using GIS technology. In our preliminary analysis of data collected through the MDSS, about 45% of *Salmonella *infections cases in Michigan are reported from a tri-county area in southeast Michigan, which contains a large portion of the Metro-Detroit area. The current analysis was restricted to this tri-county area that includes Wayne, Oakland, and Macomb Counties and accounts for about 40% of the total Michigan population.

### Data sources and database development

Variables for the database were collected from the following data sources:

### i) Michigan Disease Surveillance System

Salmonellosis is included in the Michigan Communicable Diseases Rule as a notifiable disease [22]. Physicians and laboratories are required to report cases of salmonellosis to local health departments (LHDs). LHDs investigate suspected cases of salmonellosis and collect patients' demographic (age, sex, race, and area of residence) and food history data for submission to the Michigan Department of Community Health (MDCH) through the MDSS. MDSS is a centralized, statewide, web-based database of reportable diseases. In addition to reporting to MDCH, LHDs also send patients' clinical specimens to the Bureau of Laboratories, MDCH for confirmation and serotyping. Patients' demographic and clinical specimen data are linked in the MDSS.

### ii) United States Bureau of Census

The U.S. Bureau of Census is required by constitution to conduct a population census every ten years (decennial census). The most recent census was carried out in April 2000. The Bureau collects data on various socioeconomic (e.g., household income, family size) and demographic (e.g., area of residence) attributes from every household in the U.S. and its territories. Additionally, census data are available for many levels of geography, including states, counties, cities and towns, ZIP codes, census tracts, and block groups. Block groups (prior to 2000, called a block numbering area) were chosen as the unit of analysis in this research. A block group, the smallest geographic census unit for which census socioeconomic and demographic data are available for public use, is a subdivision of a census tract and is defined as a small geographic area with a population of about 1,000 individuals, which is a relatively permanent statistical subdivision of a census tract designed to be relatively homogenous with respect to population characteristics, economic status, and living conditions [23].

A database containing SES characteristics and aggregated number of cases of salmonellosis by block group was developed using a Microsoft Excel spreadsheet (MS, 2003, Redmond, WA) and then exported it to ArcGIS (version 9.2) for geocoding.

#### Geocoding and spatial analysis

Each individual case's residential address was geocoded using Environmental Systems Research Institute's (ESRI) ArcGIS software. Geocoding is an automated process where a GIS will assign geographic coordinates to a given address based on a reference network of roads that includes information such as road names, address ranges, ZIP codes, or other geographic identifiers. The reference network used was the latest version of StreetMap U.S.A, which was included with ArcGIS 9.2. The geocoding engine used the StreetMap U.S.A Composite Locator and the default program settings for the geocoder were used (spelling sensitivity score of 80, minimum candidate score of 10, and minimum match score of 70) during the automated geocoding process. The case locations were joined with selected socioeconomic characteristics at the block group level to study the distribution of salmonellosis cases relative to the variables under investigation. The linked (SES variables and case locations) block group level data were then imported into SAS software (version 9.1) for data management and statistical analyses.

#### Data management

##### Variable selection and transformation

###### Household

According to U.S. Bureau of Census, a household includes all the people who occupy a housing unit as their usual place of residence.

###### Age

For the analyses, the median age of the block group level was used. The individual ages used to calculate the median age at the block group level were obtained from the 2000 Census and represent a person's age as of April 1, 2000 as reported by the individual and calculated by their reported birth date.

###### Education

Education refers to the highest level of schooling completed in the population aged ≥ 25 years. We categorized block groups into low (largest proportion having no education to less than a high school degree), medium (largest proportion having at least high schooling degree and/or some college but no four year college degree), and high educational attainment (largest proportion having at least a four year college degree).

###### Household income

Total income of the household is the sum of the amounts reported separately for wages, salary, commissions, bonuses, self-employment, and any public assistance or welfare payments from the state or local welfare office, including unemployment compensation. We obtained the median income of households by block group from the census data. We divided block groups into four groups based on the quartiles of the distribution of median income by block group.

###### Race

Race was self-reported by respondents according to the race with which they most closely identify. If an individual did not provide a race response, the race of the head of the household was assigned. Based on the race information in the Census data, we categorized block groups into predominantly White (where Caucasians were a larger proportion than other racial groups), predominantly Black (where African-Americans were a larger proportion than other racial groups), and predominantly Other (where racial groups other than Caucasians and African-Americans, e.g., Middle Eastern, Hispanic, etc., formed the largest proportion).

###### Urban, rural, or urban-rural mixed block group

An area consisting of a central place(s) and adjacent territory with a general population density of at least 1,000 people per square mile of land area is defined as urban area. Territory, population, and housing units not classified as urban are referred to as rural. Rural territory could be in metropolitan or non-metropolitan areas [23]. In our data, housing units were mainly classified under urban and few were categorized as rural block groups. Other block groups had both urban and rural areas and were classified as urban-rural mixed block groups.

### Dependant variable

Laboratory confirmed *Salmonella *infections cases of human origin reported to MDCH via MDSS from January 1, 1997 through December 31, 2006 were included in this study. The number of reported cases was summed by block group.

### Statistical analysis

Since our outcome variable was 'count of cases' in each block group, which ranged from 0 to 11 cases, we used Poisson regression analysis to study associations between predictor variables and the outcome [24]. Univariate analyses were performed to evaluate associations between independent variables and *Salmonella *infections. A multivariate model was developed to study the individual effect of each socioeconomic variable and *Salmonella *infections after controlling for potential confounders (median age and average household size at the block group level). Measures of association were expressed as rate ratios (RRs) with 95% confidence intervals (CIs). Besides main effects, we evaluated the interactions between race and education and race and income. To account for the difference in the population sizes of block groups, we used the 'offset' option in the proc genmod statement of SAS, which accounts for a denominator when computing the incidence. Additionally, we performed a 'log transformation' of the block group population using the proc genmod statement.

It should be noted that socioeconomic characteristics of any population could not be completely independent of each other [25]. However use of a multivariate model for identification of factors that predict differences in rates of salmonellosis, statistically adjusts for the correlation among these variables and the effects observed are therefore independent of any correlation. A p-value of < 0.05 was considered statistically significant in all analyses.

#### Ethical considerations

Cases were de-identified (personal identifiers such as names and residential addresses were removed) and only group level data were used for this analysis. The maps provide a visualization of case-density within block groups and do not represent actual case locations. This study was reviewed and approved by the Institutional Review Boards (IRBs) at Michigan State University and MDCH.

## Results

The tri-county area of Michigan (Wayne, Oakland, and Macomb) consisted of 3,604 block groups. To remove outliers from the dataset and obtain meaningful estimates, we excluded 185 scarcely populated (population < 500) block groups. Among the excluded block groups, 46 were non-residential block groups with zero population. The total population in the excluded block groups was 55,058, which represents 1.36% of the total tri-county population. The final analysis was thus performed using the data on 3,419 block groups comprising a population of 3,987,885.

**Figure 1:**
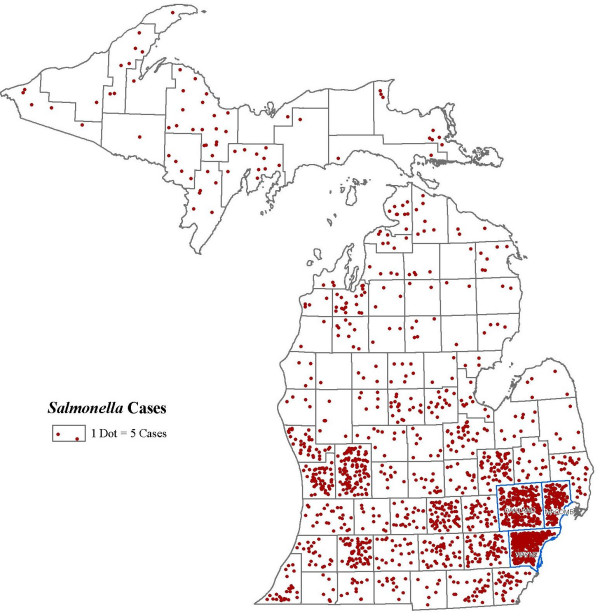
***Salmonella *infections cases by county, Michigan**. Distribution of the cases of *Salmonella *infections by county, Michigan, 1997–2006.

**Figure 2:**
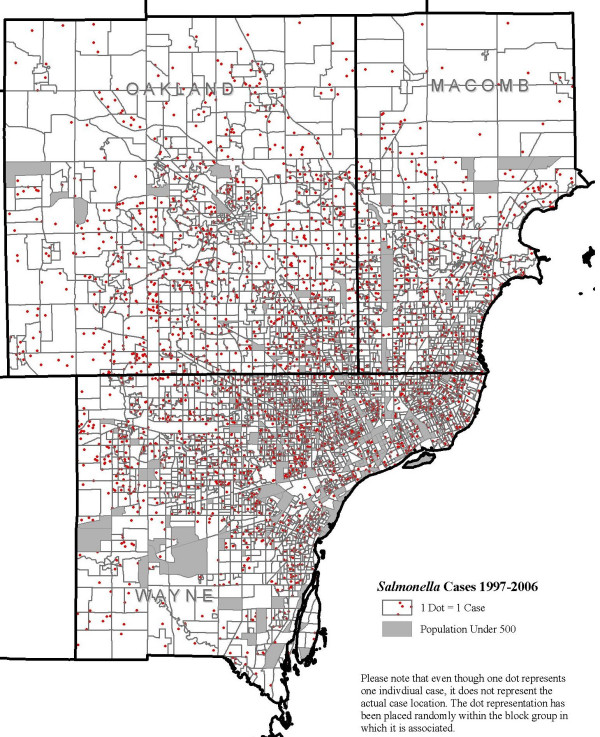
***Salmonella *infections cases by block group, Michigan**. Distribution of the cases of *Salmonella *infections by block group in Wayne, Oakland and Macomb counties, Michigan, 1997–2006.

Figure. 1 shows the distribution of the cases of salmonellosis across Michigan by county. A large number of cases in Wayne, Macomb and Oakland counties can be seen when compared to other counties in Michigan. Illustration of case distribution at the block group level in these three counties does not show any visually distinct case distribution pattern (Figure 2).

**Figure 3:**
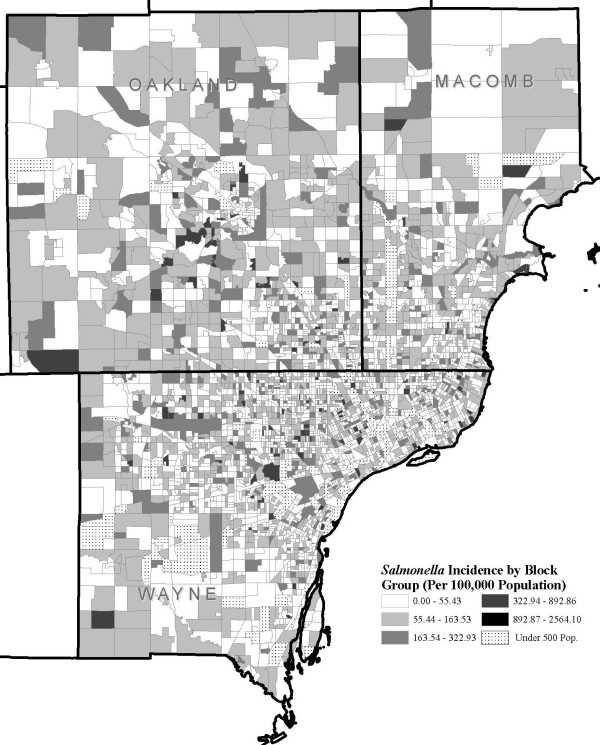
***Salmonella *incidence in Wayne, Oakland and Macomb, Michigan**. *Salmonella *infections incidence per 100,000 population by block group in three counties in Wayne, Oakland and Macomb counties, Michigan, 1997–2006.

**Figure 4:**
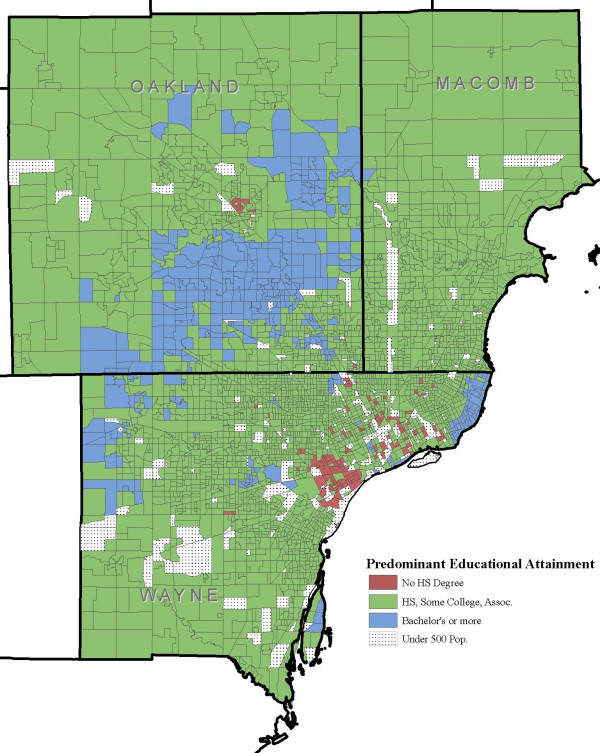
**Education attainment at the block group level in Wayne, Oakland and Macomb, Michigan**. Distribution of block groups by education attainment at the block group level in Wayne, Oakland and Macomb counties, Michigan.

**Figure 5:**
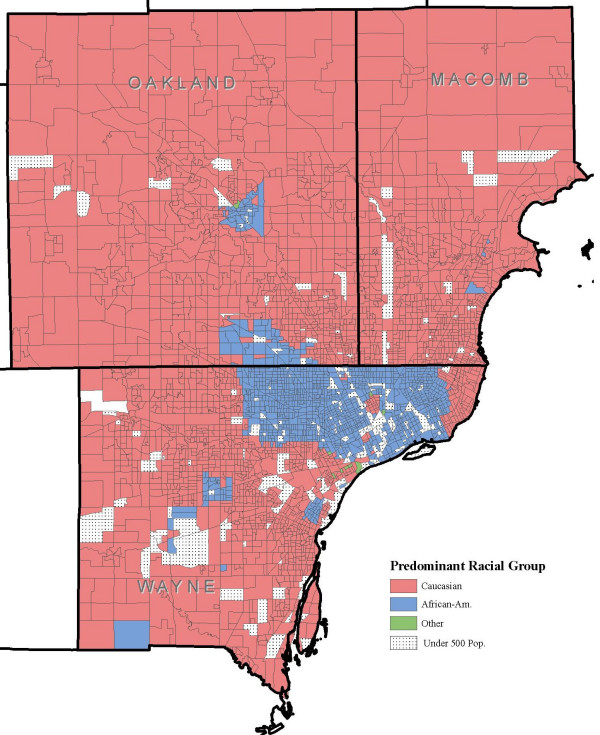
**Racial distribution at the block group level in Wayne, Oakland and Macomb, Michigan**. Distribution of block groups by race at the block group level in Wayne, Oakland and Macomb counties, Michigan.

**Figure 6:**
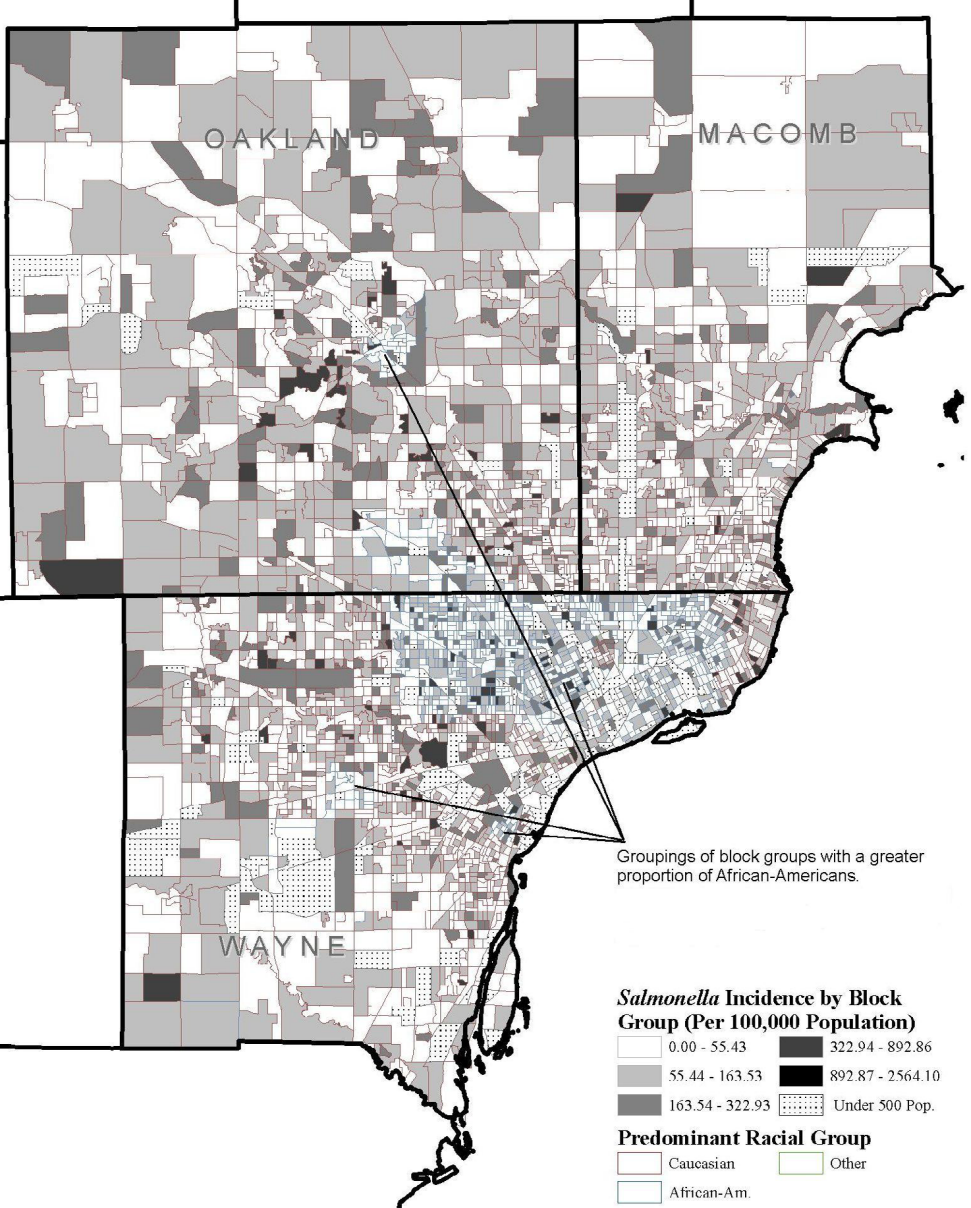
**Salmonella incidence by education attainment at the block group level**. *Salmonella *infections incidence per 100,000 population by education attainment at the block group level in Wayne, Oakland and Macomb counties, Michigan, 1997–2006.

**Figure 7:**
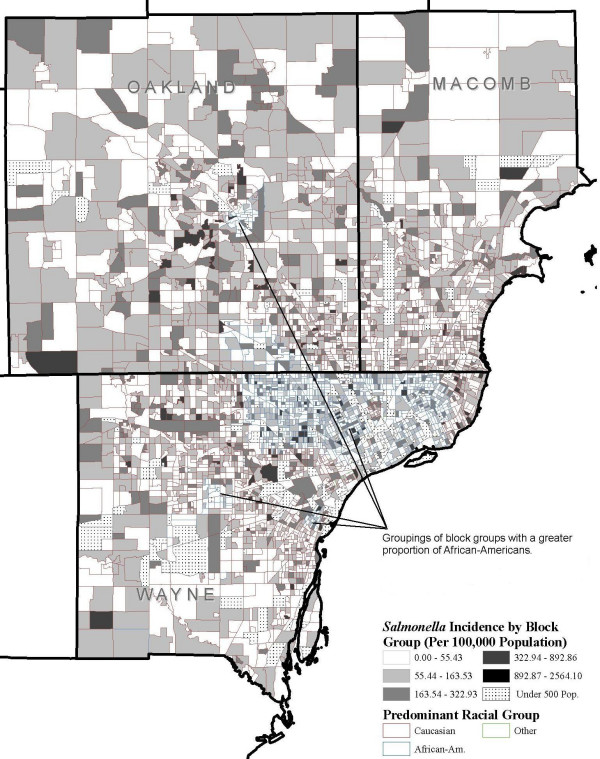
**Salmonella incidence by race at the block group level**. *Salmonella *infections incidence per 100,000 population by race at the block group level in Wayne, Oakland and Macomb counties, Michigan, 1997–2006

Figure. 3 shows *Salmonella *incidence per 100,000 population at the block group level, and Figures 4 and 5 show the distribution of block groups by education attainment and race in Wayne, Macomb and Oakland counties, respectively. These maps show what appears to be grouping within educational attainment and race. There also seems to be grouping of block groups by *Salmonella *infections incidence, as shown in Figure 3, but it is less clear and not to the extent seen in Figure 4 and Figure 5. We used the word 'grouping' and not 'cluster' because these groups were identified only by visual association and not through any type of cluster detection routines or algorithms. Additionally, when the maps from education attainment (Figure. 4) and race (Figure. 5) were overlaid onto *Salmonella *incidence map (Figure. 3) as seen in Figure 6 and Figure 7 respectively, some patterns emerge that support our statistical findings as shown Table 1.

**Table 1:** SES characteristics and *Salmonella *infection at the block group level. Socioeconomic and demographic predictors of *Salmonella *infections at the block group level in a tri-county area of Michigan (1997–2006): results of a multivariable Poisson regression model. (n = 3,419)

**Variable**	**Block groups # (%)**	**Un-adjusted RR (95% CI)**	**Adjusted RR (95% CI)**
Education attainment (aged ≥ 25 years) at block group level			
College degree and above	581 (16.99)	Reference	Reference
High school degree, some college education but no degree	2667 (78.01)	0.79 (0.73, 0.86)	0.84 (0.76, 0.92)*
None to less than a high school degree	171 (5.00)	0.74 (0.61, 0.90)	0.71 (0.65, 1.00)*

Annual median household income at block group level (highest to lowest)			
4^th ^quartile (≥ $ 60,000)	965 (28.22)	Reference	Reference
3^rd ^quartile ($ 47,000 – < $60,000)	734 (21.47)	0.94 (0.86, 1.35)	0.99 (0.89, 1.10)
2^nd ^quartile ($33,000 – < $ 47,000)	880 (25.74)	0.80 (0.73, 0.88)	0.86 (0.77, 0.97)*
1^st ^quartile (< $ 33,000)	840 (24.57)	0.82(0.75, 0.91)	0.91 (0.79, 1.04)

Race at block group level (based on the largest proportion)			
Caucasian, predominantly	2337 (68.35)	Reference	Reference
African American, predominantly	997 (29.16)	0.87 (0.80, 0.95)	0.94 (0.84, 1.05)
Other, predominantly	85 (2.49)	0.91 (0.73, 1.13)	0.96 (0.76, 1.22)

Area of residence at block group level (based on US Bureau of Census definition)			
Rural, predominantly	27 (0.79)	Reference	Reference
Urban-rural mixed	107 (3.13)	1.18 (1.30, 1.80)	1.13 (0.74, 1.74)
Urban, predominantly	3285 (96.08)	1.15 (0.77, 1.74)	1.19 (0.79, 1.78)

*Significant at 0.05 in the adjusted model; RR: rate ratios; CI: confidence interval.

Final Poisson regression model was adjusted for median age, median income, education level, race, and area of residence at the block group level

Table 1 shows the results of univariate and multivariate Poisson regression models. The final multivariate model revealed that block groups with lower educational attainment were less commonly represented among cases than their counterparts with higher education levels (< high school degree vs. ≥ college degree: rate ratio (RR) = 0.79, 95% confidence interval (CI):0.63, 0.99; and high school degree and above but no college degree vs. ≥ college degree; RR = 0.84, 95% CI: 0.76, 0.92). Levels of education also showed a dose-response relation with the outcome variable, i.e., decreasing years of education at the block group level was associated with a decrease in the incidence of *Salmonella *infections. Race and area of residence were not associated with *Salmonella *infections at the group level. Moreover, interactions between 'income and race' and 'education and race' were not statistically significant.

## Discussion

In this study using spatial geo-referenced data, we visualized, explored, and modeled the associations between selected socioeconomic and demographic attributes from by the U.S. Bureau of Census and reported *Salmonella *infections in three large counties in Michigan. GIS is emerging as an efficient tool to support the results of traditional descriptive epidemiology–it allows researchers to hypothesize meaningful associations from the spatial data, identify high risk areas, and help guide future research. We found a concentration of block groups with a higher proportion of educated individuals in the southern part of Oakland and eastern part of Wayne counties. A higher incidence of salmonellosis was seen in the block groups with high education compared to the less educated block groups (Figure 6). A large grouping of block groups with a high proportion of African-Americans was found in the Detroit metropolitan area.

Socioeconomic and demographic indicators can be used to predict which individuals and communities are at an increased risk of acquiring infections. Generally, low SES is an important predictor of several poor health outcomes including chronic diseases, mental illnesses, and mortality [11,26]. However, our multivariate model revealed a higher rate of reported *Salmonella *infections in block groups where a greater proportion of individuals with high educational attainment resided.

No prior studies using group level data investigated the relation between SES and salmonellosis. However, using a spatial analysis technique similar to our study, Green et al. [27], found a positive association between enteric campylobacter infections and higher SES status in Manitoba, Canada (1996–2004). This greater incidence of *Campylobacter *infection for individuals of higher SES status compared to their counterparts was partially attributed to increased opportunities for foreign travel to areas where *Campylobacter *infection is endemic and frequent consumption of food prepared outside the home [27]. Similar risk factors for *Salmonella *infections have been reported in other individual level epidemiologic investigations [28,29]. In addition to these reported risk factors for salmonellosis, our findings may be explained in part by greater access to healthcare for those in the high education and income groups, which would increase the detection of *Salmonella *cases. Mead et al. [5] stated that only a small fraction of individuals with *Salmonella *infection are diagnosed and reported [30]. It is possible that a large proportion of unreported cases, particularly the relatively less severe episodes, among residents of low education-block groups who often do not have health insurance, go unreported because they do not seek medical care that would lead to specimen collection, diagnosis, and reporting. This is corroborated by the findings from a recent study that revealed that cases of acute diarrheal illness ascertained through laboratory-based public health surveillance differ systematically from unreported cases by the health insurance factor [30]. Similarly, residents of high education block groups may seek medical consultation even for mild to moderate symptoms of enteric infections, including salmonellosis, thus increasing their likelihood of becoming a reported case. In contrast, individuals in block groups having low education may tend to ignore mild symptoms of the disease, resulting in a larger proportion of unreported cases being missed by the existing laboratory-based passive surveillance system.

In addition, individuals of higher education block groups who also have greater discretionary income may eat outside the home frequently and be more likely to own pets considered reservoirs of *Salmonella*, which increase the likelihood of contracting *Salmonella *infections compared to their counterparts with lower levels of education. In a cross-sectional study based on a sample of veterinary hospital clients in Utah about 77% of exotic pet (e.g., ferrets, lizards, turtles) owners had at least some college education and that their mean family income ranged between $35,000 and $50,000 per year [31]. The limited purchasing power and access to supermarkets and pet stores may reduce the exposure of lower SES and educational status populations to foods and pets that are frequently associated with salmonellosis.

Since income and education generally have a strong positive correlation [25], which is shown in our data as well (p-value < 0.01), we expected a similar effect of income on the rate of *Salmonella *infections at the block group level. However, in our final Poisson regression model, levels of income at the block group level did not demonstrate a consistent dose response gradient with the outcome variable as depicted in the case of levels of education. An explanation for this observation may be that education itself has a strong effect on health seeking behavior irrespective of the income level – better educated people are more likely to seek medical treatment than less educated individuals.

In recent years, racial disparities in healthcare in the U.S. have been a major focus in epidemiologic research [19]. Although data on African-American and Caucasian differences in mortality from infectious diseases are available [32], few studies have investigated differences in food borne infections between Caucasians and other racial minorities [33,34]. The incidences of food borne infections, including salmonellosis, may differ across racial and ethnic groups due to variations in food preferences, preparation methods, and handling among racial groups [33,35]. However limited data exist to delineate specific food preparation and handling methods responsible for the acquisition of enteric infections for specific racial subgroup populations.

In the year 2000, a significantly higher rate of *Salmonella *infections among African-Americans compared to Caucasians was reported [36]. In addition, Marcus et al. (2007) found the highest average annual incidence (1998–2000) of *Salmonella *serotype Enteritidis in African-Americans (2.0/100,000), followed by Hispanics (1.2/100,000), and Caucasians (1.1/100,000) [37]. In contrast to the findings based on individual level studies, our group level data analysis did not find an association between race at the block group level and *Salmonella *infections.

In accordance with our previous individual level studies using Michigan data [17,18], we did not find an association between *Salmonella *infections and urban or rural block groups. This suggests that populations in both of these settings are exposed to similar levels of potential sources of *Salmonella *infections.

Inherent limitations of GIS based data and our analysis should be considered when interpreting the results [20,38]. We used the block group as the unit of analyses, but analyses based on other census geography (e.g., census tract or county) may provide different results. In any group level data, variations within the group are masked, limiting researchers' ability to study any differences within the unit of analysis (in our case block group). Additionally, the fact that individual level studies usually utilize more complete data on race and socioeconomic status cannot be overlooked. Since our study used a group level analysis, attempts to draw individual level inferences is inappropriate and may lead to a biased interpretation (ecological fallacy) [20]. Our case data spanned the years from 1997 to 2006. However, we used the Census 2000 200 data for analyses in order to approximate a middle point between the years of the study, which would and minimize the effects of any population change that occurred over the study period. The Census data is also the best freely available, standard, population dataset that provided robust data on the scale levels that we wished to conduct our research. As mentioned earlier, we excluded 185 block groups with < 500 population from our analysis. Since population of excluded block groups accounted for 1.36% of the total population in the three county area, we believe that exclusion of these scarcely populated block groups have not effected our results.

The spatial aspects of this study are somewhat limited due to the scope of the study. Our primary objective was to study the association between socioeconomic variables (which were not available in the surveillance database) and reported cases of *Salmonella *infections using GIS technology. It should be noted that the spatial join used in this study can be done without the use of GIS, however, visualization of the data using maps allows researchers to examine visual association between the variable of interest and outcome in addition to statistical analyses.

## Conclusion

We have used GIS technology to study associations between SES attributes and salmonellosis in the three most populated counties in Michigan. Our results suggest that education may play a significant role in health-seeking behavior and the predisposition for *Salmonella *infections at the population level. The results are different from reported individual level epidemiologic studies that have found a higher level of foodborne infections among low education and low income groups. This apparent discrepancy may be explained because individuals of higher income block groups might eat *Salmonella*-contaminated foods more frequently and be more likely to own *Salmonella*-reservoir pets, which increases the likelihood of contracting *Salmonella *infections compared to their counterparts with lower levels of education. It is also conceivable that reporting bias exists due to a greater detection of *Salmonella *infections among high education block groups compared to low education block groups. Since under-reporting is observed mainly in less educated areas, efforts are needed to increase case detection from such localities. In addition, this analysis demonstrates that GIS is a useful tool in epidemiologic research for exploring associations between neighborhood level characteristics and the distribution of infectious agents like *Salmonella*.

## Competing interests

The author(s) declare that they have no competing interests.

## Authors' contributions

All authors have read and approved the final manuscript.

MY: Major role in study conception, design, data collection, analyses, and writing of the manuscript

EH: Developing maps and data linking using the GIS technology

AAS: Statistical analyses of data and manuscript writing

MW: Coordinating the data acquisition and critical review of the manuscript

HDD: Contributing to the design and writing of the manuscript

MR: Statistical consultant and contributor to the writing of the manuscript

JF: Advice and critical review of the manuscript

AMS: Supervision of the entire study, advice, writing contributor, and support of the project.

## References

[B1] Olsen SJ, Bishop R, Brenner FW, Roels TH, Bean N, Tauxe RV, Slutsker L (2001). The changing epidemiology of salmonella: trends in serotypes isolated from humans in the United States, 1987-1997. J Infect Dis.

[B2] Center for Disease Control and Prevention (2006). Preliminary FoodNet Data on the Incidence of Infection with Pathogens Transmitted Commonly Through Food, 10 States, United States.. Morb Mortal Wkly Rep.

[B3] Angulo FJ, Voetsch AC, Vugia D, Hadler JL, Farley M, Hedberg C, Cieslak P, Morse D, Dwyer D, Swerdlow DL (1998). Determining the burden of human illness from food borne diseases. CDC's emerging infectious disease program Food Borne Diseases Active Surveillance Network (FoodNet). Vet Clin North Am Food Anim Pract.

[B4] Voetsch AC, Van Gilder TJ, Angulo FJ, Farley MM, Shallow S, Marcus R, Cieslak PR, Deneen VC, Tauxe RV (2004). FoodNet estimate of the burden of illness caused by nontyphoidal Salmonella infections in the United States. Clin Infect Dis.

[B5] Mead PS, Slutsker L, Dietz V, McCaig LF, Bresee JS, Shapiro C, Griffin PM, Tauxe RV (1999). Food-related illness and death in the United States. Emerg Infect Dis.

[B6] Trevejo RT, Courtney JG, Starr M, Vugia DJ (2003). Epidemiology of salmonellosis in California, 1990-1999: morbidity, mortality, and hospitalization costs. Am J Epidemiol.

[B7] Schutze GE, Kirby RS, Flick EL, Stefanova R, Eisenach KD, Cave MD (1998). Epidemiology and molecular identification of Salmonella infections in children. Arch Pediatr Adolesc Med.

[B8] Chan-yeung M, Yeh AG, Tam CM, Kam KM, Leung CC, Yew WW, Lam CW (2005). Socio-demographic and geographic indicators and distribution of tuberculosis in Hong Kong: a spatial analysis. Int J Tuberc Lung Dis.

[B9] Everson SA, Maty SC, Lynch JW, Kaplan GA (2002). Epidemiologic evidence for the relation between socioeconomic status and depression, obesity, and diabetes. J Psychosom Res.

[B10] Koster A, Penninx BW, Bosma H, Kempen GI, Harris TB, Newman AB, Rooks RN, Rubin SM, Simonsick EM, van Eijk JT, Kritchevsky SB (2005). Is there a biomedical explanation for socioeconomic differences in incident mobility limitation?. J Gerontol A Biol Sci Med Sci.

[B11] Martikainen P, Stansfeld S, Hemingway H, Marmot M (1999). Determinants of socioeconomic differences in change in physical and mental functioning. Soc Sci Med.

[B12] Liu L, Deapen D, Bernstein L (1998). Socioeconomic status and cancers of the female breast and reproductive organs: a comparison across racial/ethnic populations in Los Angeles County, California (United States). Cancer Causes Control.

[B13] Ronald LA, Kenny SL, Klinkenberg E, Akoto AO, Boakye I, Barnish G, Donnelly MJ (2006). Malaria and anaemia among children in two communities of Kumasi, Ghana: a cross-sectional survey. Malar J.

[B14] Akbar N, Basuki B, Garabrant DH, Sulaiman A, Noer HM, Mulyanto (1997). Ethnicity, socioeconomic status, transfusions and risk of hepatitis B and hepatitis C infection. J Gastroenterol Hepatol.

[B15] Krieger N (1992). Overcoming the absence of socioeconomic data in medical records: validation and application of a census-based methodology. Am J Public Health.

[B16] Krieger N, Chen JT, Ebel G (1997). Can we monitor socioeconomic inequalities in health? A survey of U.S. health departments' data collection and reporting practices. Public Health Rep.

[B17] Younus M, Wilkins MJ, Arshad MM, Rahbar MH, Saeed AM (2006). Demographic risk factors and incidence of Salmonella enteritidis infection in Michigan. Foodborne Pathog Dis.

[B18] Arshad MM, Wilkins MJ, Downes FP, Rahbar MH, Erskine RJ, Boulton ML, Saeed AM (2007). A registry-based study on the association between human salmonellosis and routinely collected parameters in Michigan, 1995-2001. Foodborne Pathog Dis.

[B19] Department of Health and Human Services (2000). Healthy people 2010: Understanding and improving health.. US Government Printing Office, Washington DC.

[B20] Vine MF, Degnan D, Hanchette C (1997). Geographic information systems: their use in environmental epidemiologic research. Environ Health Perspect.

[B21] Ruankaew N (2005). GIS and epidemiology. J Med Assoc Thai.

[B22] Michigan Department of Community Health (2001). Health care professionals' guide to the Michigan communicable disease rules..

[B23] US Department of Commerce (2000). Census 2000 geographic terms and concepts..

[B24] Kleinbaum DG (1998). Applied Regression Analysis and Other Multivariable Methods.. Duxbury Press Pacific Grove, California, USA.

[B25] Merkin SS, Diez Roux AV, Coresh J, Fried LF, Jackson SA, Powe NR (2007). Individual and neighborhood socioeconomic status and progressive chronic kidney disease in an elderly population: The Cardiovascular Health Study. Soc Sci Med.

[B26] Smith GD, Hart C (1998). Socioeconomic factors and determinants of mortality. Jama.

[B27] Green CG, Krause D, Wylie J (2006). Spatial analysis of Campylobacter infection in the Canadian province of Manitoba. Int J Health Geogr.

[B28] Marcus R, Varma JK, Medus C, Boothe EJ, Anderson BJ, Crume T, Fullerton KE, Moore MR, White PL, Lyszkowicz E, Voetsch AC, Angulo FJ (2007). Re-assessment of risk factors for sporadic Salmonella serotype Enteritidis infections: a case-control study in five FoodNet Sites, 2002-2003. Epidemiol Infect.

[B29] Jones TF, Angulo FJ (2006). Eating in restaurants: a risk factor for foodborne disease?. Clin Infect Dis.

[B30] Scallan E, Jones TF, Cronquist A, Thomas S, Frenzen P, Hoefer D, Medus C, Angulo FJ (2006). Factors associated with seeking medical care and submitting a stool sample in estimating the burden of foodborne illness. Foodborne Pathog Dis.

[B31] Klaphake EA, Smith JL (2002). An Initial Assessment of Exotic-Animal Pet Owners in Utah: A Survey With Special Emphasis on Personal Characteristics and Expenditure Tendencies. Journal of Avian Medicine and Surgery.

[B32] Richardus JH, Kunst AE (2001). Black-white differences in infectious disease mortality in the United States. Am J Public Health.

[B33] Center for Disease Control and Prevention (1998). FoodNet presentation: age, ethnic and racial disparity in Salmonella serotype Enteritidis. FoodNet.

[B34] Center for Disease Control and Prevention (2002). Racial Disparities in Nationally Notifiable Diseases, United States, 2002.. MMWR.

[B35] Ladd-Wilson S, Yang S, Deneen V, Koehler J, Marcus R, Vugia D, Voetsch D, Angulo High-Risk Food Consumption, handling, and Preparation Practices of Adults in the FoodNet Sites, 1996-1997.. 1st International Conference on Emerging Infectious Diseases Atlanta, GA, March 1998.

[B36] Lay J VJ, Vugia D, Jones T, Zansky S (2002). Racial and Ethnic Disparties in foodborne illness, 2000.. Infectious Disease Society of America, Chicago, IL.

[B37] Marcus R RET, Lay J, Mohle-Boetani J, Farley M (2002). Age, Ethnic and Racial Disparity in Salmonella serotype Enteritidis (SE): FoodNet, 1998-2000.. International Conference on Emerging Infectious Diseases Atlanta, GA.

[B38] Boscoe FP, Ward MH, Reynolds P (2004). Current practices in spatial analysis of cancer data: data characteristics and data sources for geographic studies of cancer. Int J Health Geogr.

